# Symptomatic 10-year-old with a large right atrial aneurysm and a secundum atrial septal defect: a case report

**DOI:** 10.1186/s13256-025-05280-5

**Published:** 2025-10-08

**Authors:** Herbert Ariaka, Aisha Nakato, Twalib Aliku, Nestor Mbabazi, Rwakaryebe Mbaaga Muhoozi, Emma Ndagire, Naomi Kebba, Sulaiman Lubega, Tom Philip Mwambu, Michael Charles Oketcho

**Affiliations:** 1 Department of Cardiovascular Surgery, Uganda Heart Institute, P.O. Box 37392, Kampala, Uganda; 2 Department of Paediatric Cardiology, Uganda Heart Institute, P.O. Box 37392, Kampala, Uganda; 3 School of Medicine, Uganda Christian University, P.O. BOX 4, Mukono, Uganda

**Keywords:** Cardiac surgery, Closure, Congenital heart defect, Resection, Right atrial aneurysm

## Abstract

**Background:**

Right atrial aneurysms are rare. If left untreated, they can result in arrhythmias, heart failure, catastrophic rupture, and thromboembolic phenomena.

**Case presentation:**

We present the case of a 10-year-old African female who presented with heart failure. The patient underwent successful open-heart surgery to close the atrial septal defect, resect the right atrial aneurysm, and directly close it. Initial peripheral cannulation was performed to decompress the heart, allowing for a safe sternotomy before transitioning to aorto-bicaval cannulation.

**Conclusion:**

Surgery remains the best option for managing this condition.

## Background

Right atrial aneurysms (RAAs) are infrequent, with few reported cases. Bailey and Gilman described a successful excision of a RAA in 1953, with another case published in 1968 [1, 2]. The diagnosis can be made prenatally, in childhood, and even later in adulthood, when most cases are diagnosed. In a review of 103 cases of congenital malformations of the right atrium and the coronary sinus, 60 patients had congenital enlargements of the right atrium [3]. Estimating the actual prevalence of RAAs in the pediatric population is challenging owing to the scarcity of literature on the subject. RAAs are often associated with arrhythmias, which can lead to sudden cardiac death. Hence, early intervention is necessary, even in asymptomatic patients [2, 4]. Other potential complications of RAAs include heart failure, rupture, thrombus formation, and paradoxical emboli [2, 5]. We present the case of a 10-year-old female who exhibited signs of heart failure characterized by easy fatiguability. She was in sinus rhythm. The patient underwent closure of the secundum atrial septal defect (ASD), resection, and direct closure of the RAA.

## Case presentation

A 10-year-old African female weighing 35 kg was admitted to our hospital with a 9-year history of chest deformity and easy fatiguability for the past month. There was no history of chest trauma, chronic illnesses, or prolonged medication use.

The blood pressure was 116/62 mmHg, the heart rate was 94 beats per minute, and the oxygen saturation was 99% on room air. The jugular venous pressure was not elevated. The patient had a pectus carinatum with therapeutic marks on the anterior chest wall, a sign that the patient had received traditional medicine for the treatment of the RAA, where short skin incisions are made at the site of the lesion and herbs are smeared into them. The apex beat was in the 5th intercostal space, anterior axillary line. Heart sounds I and II were heard but muffled, with no murmur. There was a non-tender hepatomegaly 3 cm below the subcostal margin.

**Fig. 1:**
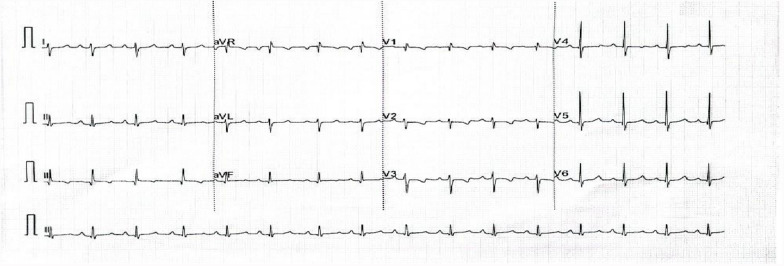
Preoperative electrocardiogram showing a sinus rhythm

**Fig. 2:**
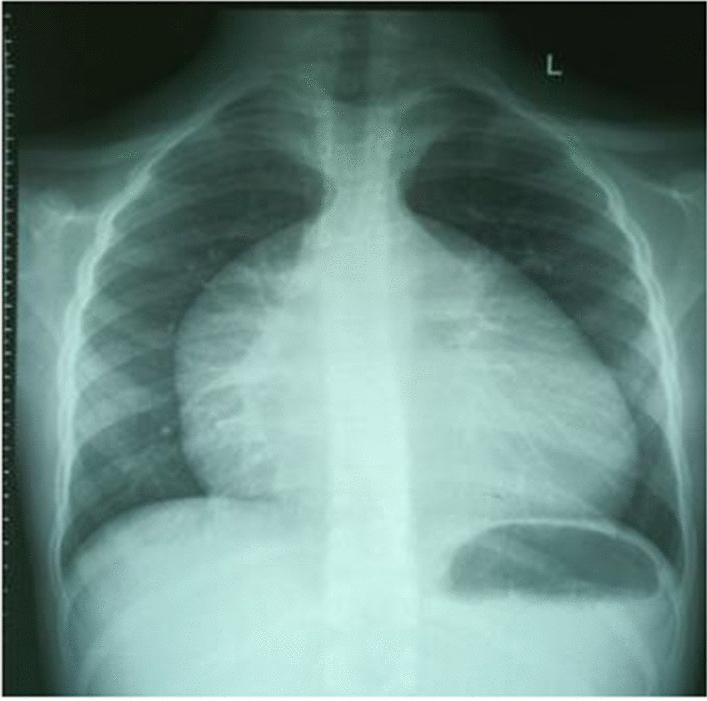
Preoperative chest X-ray showing cardiomegaly

The complete blood count (CBC), renal function tests (RFTs), and liver function tests (LFTs) were within normal limits with negative serologies for hepatitis B, C, and HIV. The preoperative electrocardiogram (ECG) (Fig. 1) showed a sinus rhythm with features of right axis deviation, right atrial enlargement, and right ventricular hypertrophy. The anteroposterior chest radiograph (Fig. 2) showed gross cardiomegaly.

**Fig. 3:**
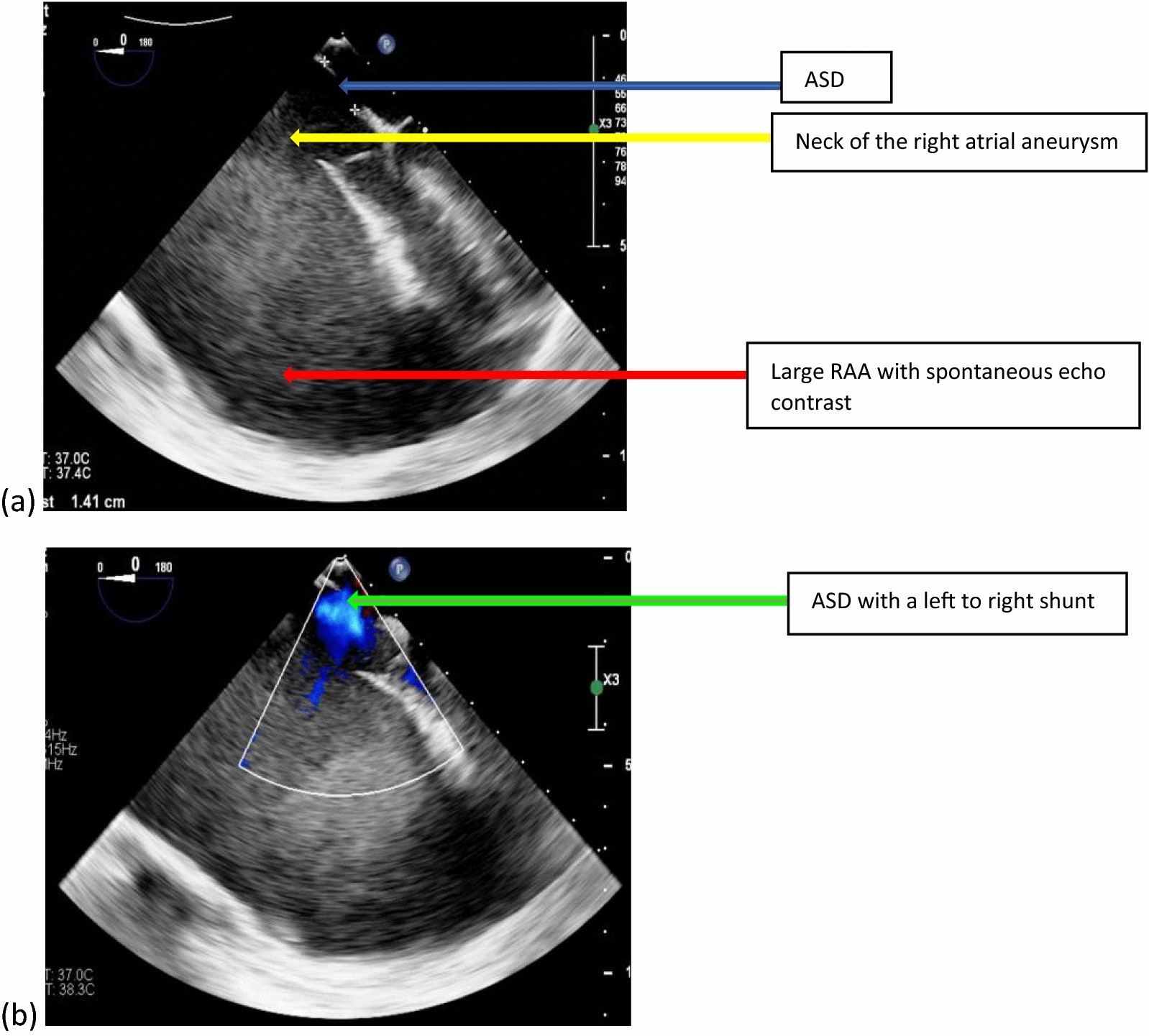
Transesophageal echocardiogram showing a large right atrial aneurysm with spontaneous echo contrast in (**a**) and a large secundum atrial septal defect in (**b**) with a left-to-right shunt

**Fig. 4:**
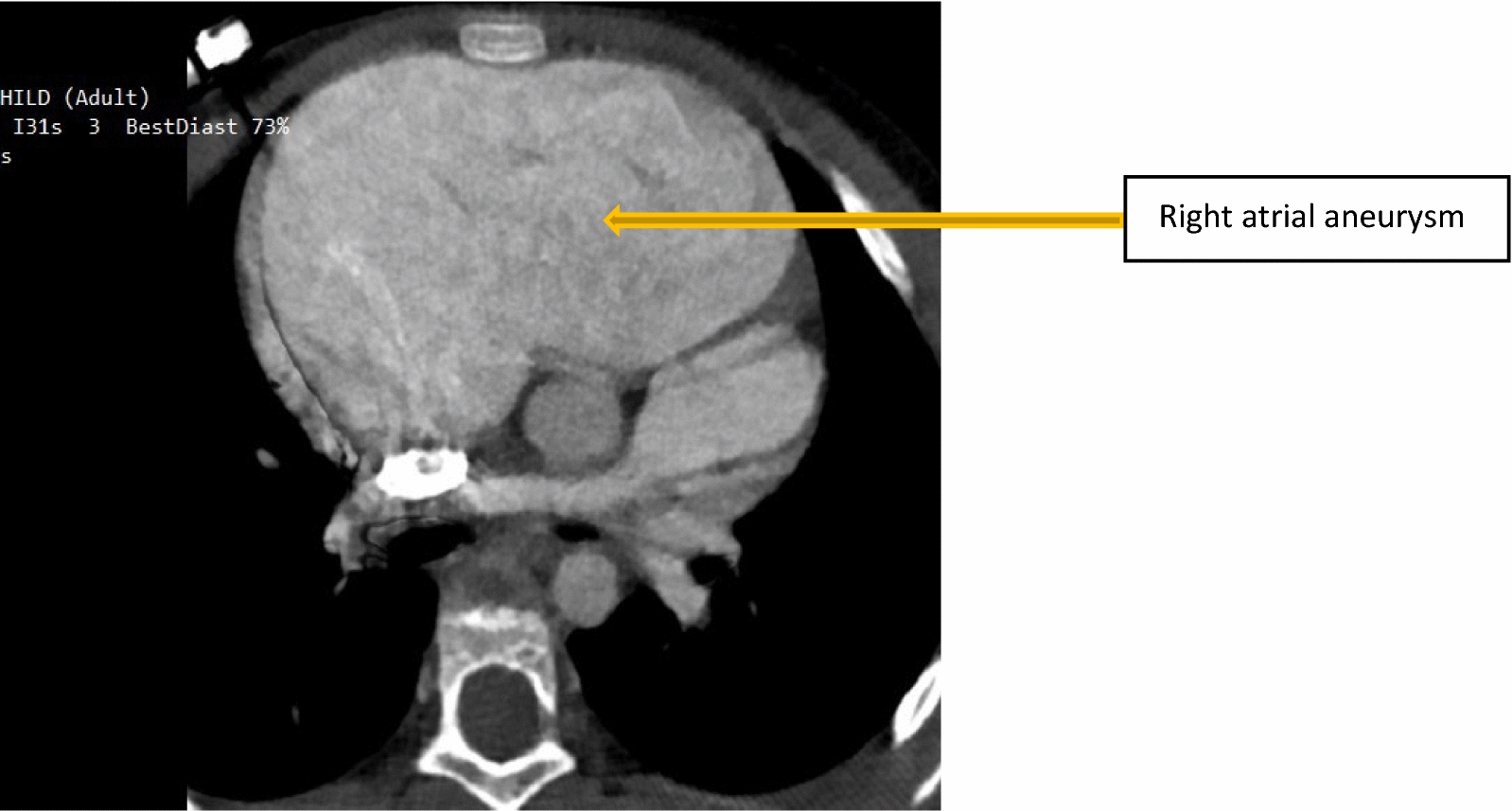
Contrast cardiac computed tomography scan showing large right atrial aneurysm

The Transesophageal echocardiogram (TEE) revealed a large right atrial aneurysm about 16 mm × 12 mm (Fig. 3a) and a large secundum ASD about 14 mm × 10 mm with a left to right shunt with normal tricuspid valve anatomy and function (Fig. 3b). All other cardiac chambers were compressed. The axial view of the contrast cardiac computed tomography (CT) showed a large RAA (Fig. 4).

### Intervention

Although the neck veins in this patient were not visibly distended during her physical examination, the internal jugular vein was grossly dilated, and spontaneous echo contrast was observed during ultrasound-guided placement of the central venous line.

**Fig. 5:**
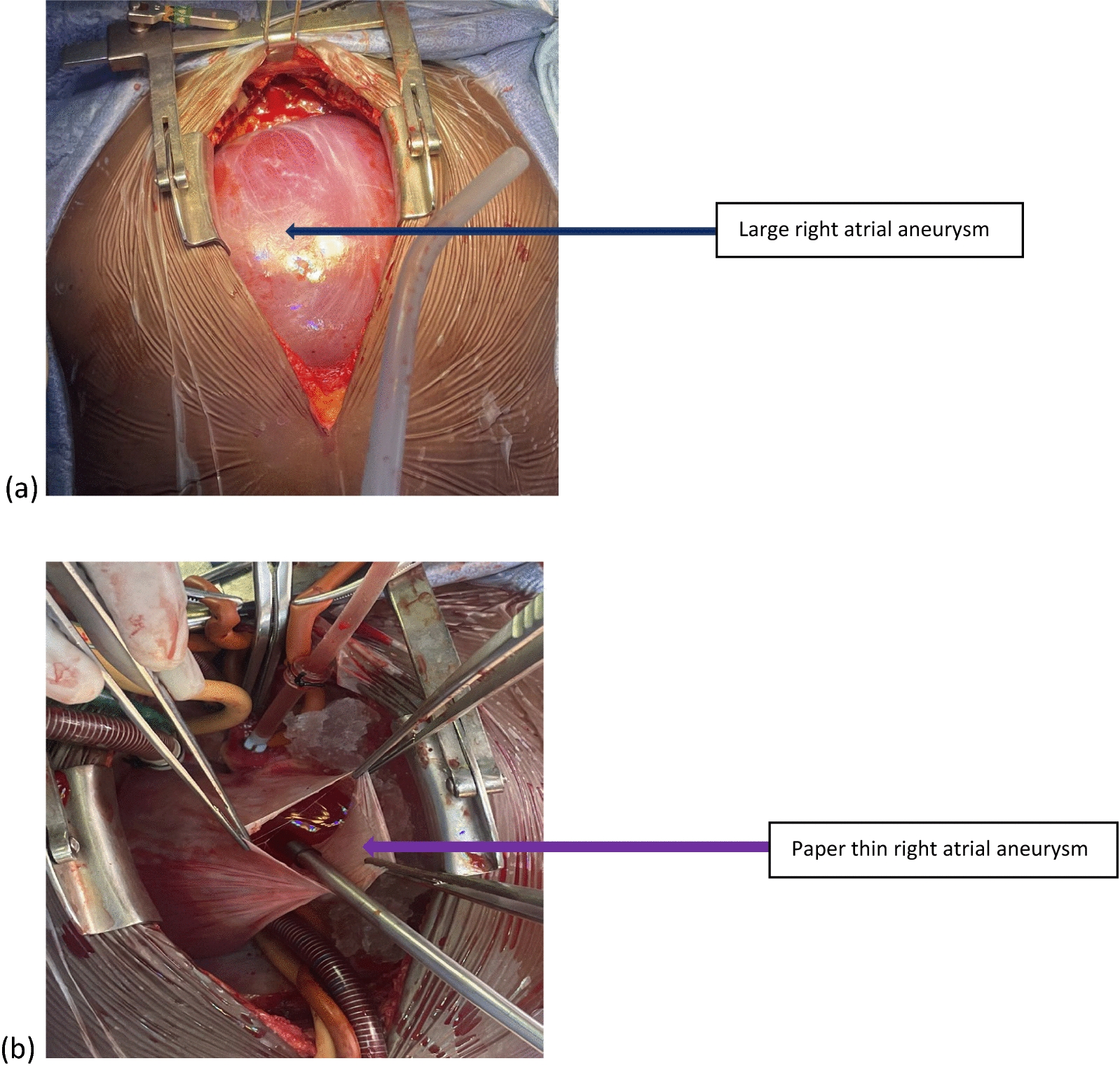
**a** Large paper-thin right atrial aneurysm occupying most of the anterior aspect of the mediastinum. **b** Incised edges of the right atrial aneurysm

**Fig. 6:**
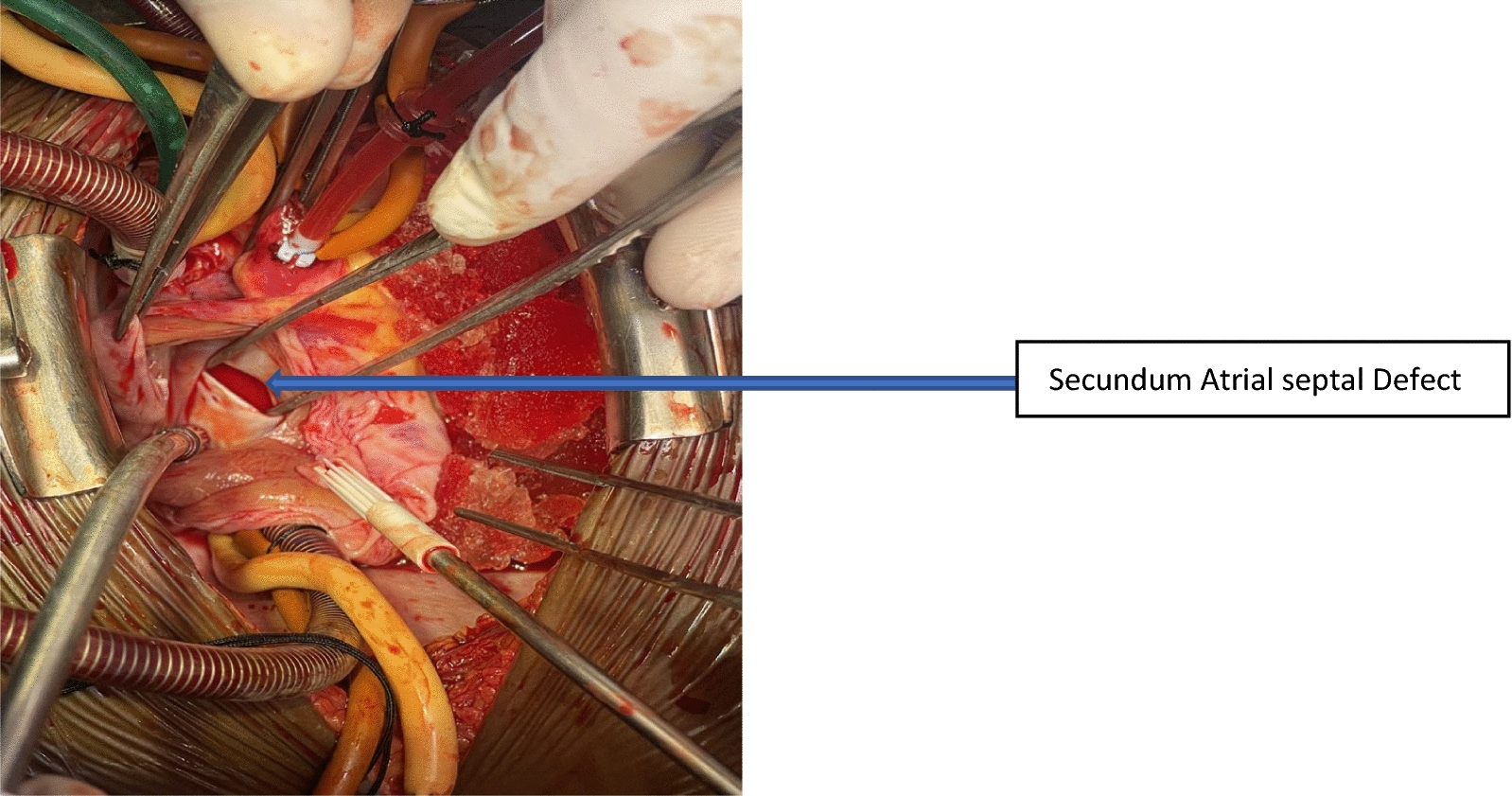
The large secundum atrial septal defect before closure

Following femoro-femoral cardiopulmonary bypass (CPB) to decompress the heart, a median sternotomy was performed. Aorto-bicaval cannulation was performed and mild hypothermia was initiated on cardiopulmonary bypass. Cold blood cardioplegia was delivered antegrade via the aortic root to achieve a diastolic arrest of the heart. Opening the pericardium and later the right atrium revealed a large paper-thin right atrial aneurysm, a normal tricuspid valve (Fig. 5a and b), and a large secundum ASD about 14 mm × 10 mm in diameter with a normal right coronary artery anatomy (Fig. 6).

**Fig. 7:**
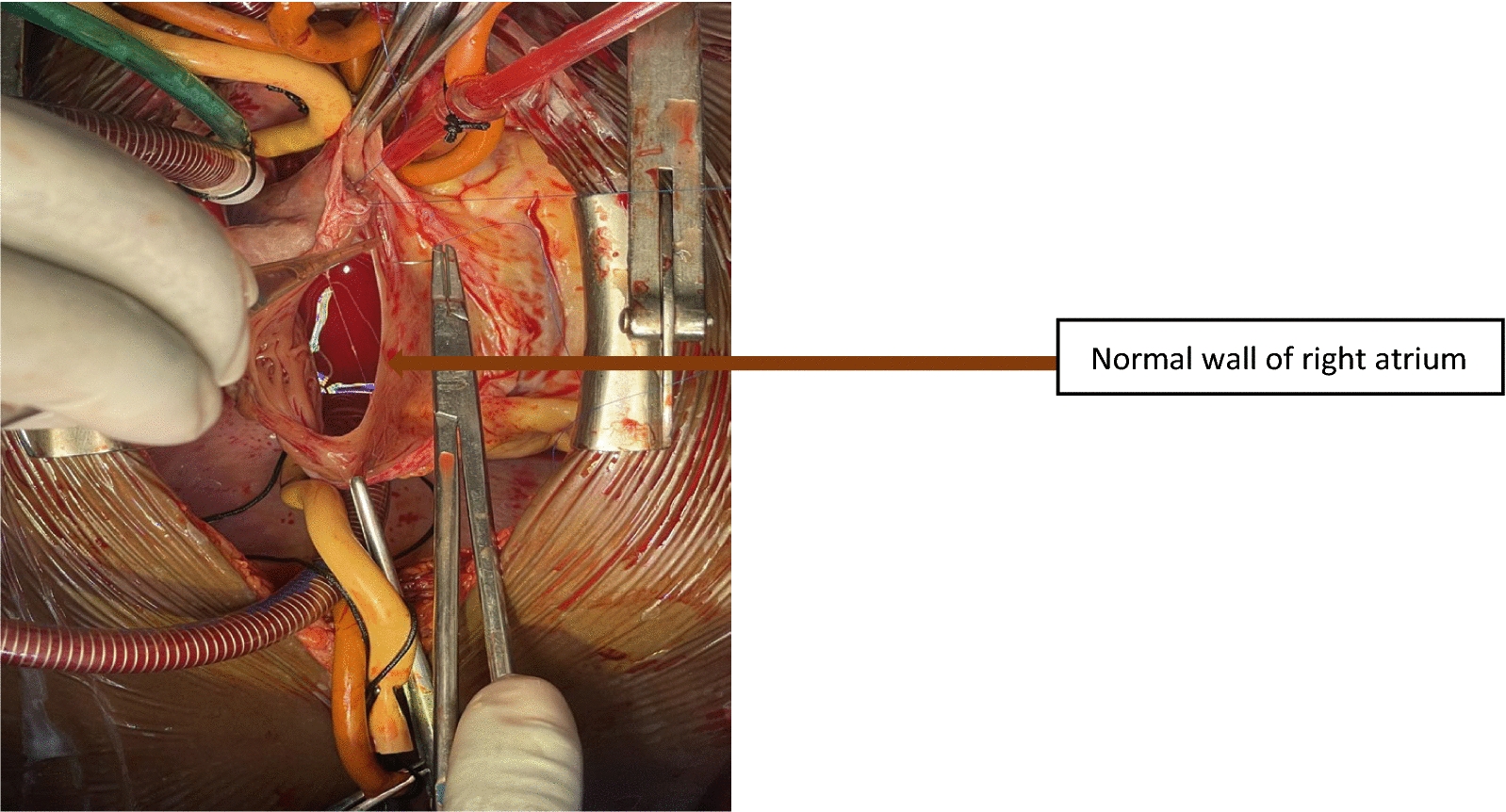
Closure of the true right atrial wall after excising the right atrial aneurysm

The ASD was closed directly using a continuous stitch because it was slit-like. Subsequently, the right atrial aneurysmal wall was resected, and the normal right atrial wall was closed directly in a double layer (Fig. 7). Hemostasis was achieved, the pericardium was approximated over the aorta, and the chest was closed in layers.

**Fig. 8:**
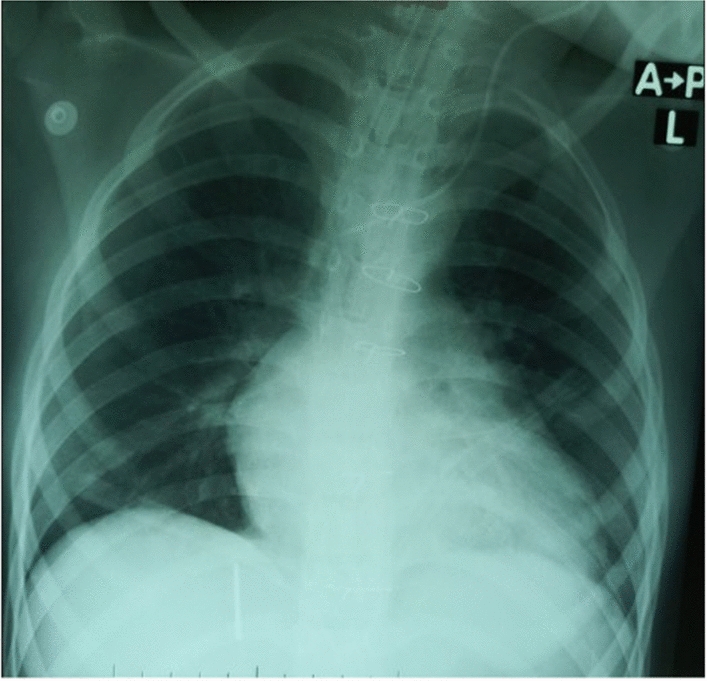
Normal cardiothoracic ratio on postoperative chest X-ray

The patient was extubated on the first postoperative day, and the rest of her recovery was uneventful. The postoperative chest X-ray showed a normal cardiothoracic ratio (Fig. 8). The postoperative transthoracic echocardiography revealed no aneurysm, mild mitral regurgitation, and mild tricuspid regurgitation with an ejection fraction (E/F) of 59%. She was discharged on an anti-failure regimen which consisted of oral lasix given at 1 mg/kg once a day and oral captopril given at 0.3 mg/kg/dose three times a day for 1 month.

### Follow-up and outcomes

The patient is stable and shows no signs or symptoms of heart failure. The postoperative ECG shows a sinus rhythm with a heart rate of 92 beats per minute, with no significant changes observed on the echocardiogram. All operation sites have healed well. The last review done 1-year postoperatively was unremarkable and all the anti-failure regimen had been stopped.

We expect this patient to have an excellent long-term prognosis. Subsequent follow-ups will be geared toward checking for any recurrence of the RAA and looking for any arrhythmias.

## Discussion

The etiology of RAAs is uncertain. They can be classified as idiopathic, with no clear elucidated mechanism or congenital, thought to be caused by dysplasia of the muscular wall of the right atrium [6]. The RAAs can either be single or multiple [5]. Our patient had a single congenital RAA. Differential diagnoses depending on the patient’s age include Ebstein’s anomaly, pericardial cyst, cardiac tumors, pericardial effusion, and a sinus of Valsalva aneurysm [7, 8]. The resected right atrial wall has been histologically found to consist of fibrous tissue and myocardial fibers with an endothelial lining [5].

Patients may be asymptomatic or present with symptoms such as palpitations, chest pain, dyspnea, syncope, severe airway obstruction, and easy fatiguability that are associated with arrhythmias and heart failure [9]. This patient exhibited signs of heart failure despite being in a sinus rhythm without a pericardial effusion. Echocardiography and cardiac CT scan were performed to aid in diagnosing this patient. These tools provided sufficient information about the anatomy of the disease and its associated anomalies. Magnetic resonance imaging is also invaluable in patients with uncertain diagnoses [8, 10]. Angiography has been performed in one case but is no longer routinely performed.

Most approaches for RAA repair are via median sternotomy because this provides adequate exposure for the heart and other mediastinal structures, except for one reported case that was performed via a clamshell thoracotomy. The surgical treatment of RAAs using cardiopulmonary bypass is generally considered safe, with little risk of sinus node dysfunction, atrial arrhythmias, or embolic phenomena. Owing to the large RAA, this patient underwent initiation of CPB via peripheral cannulation to decompress the heart, reducing the risk of inadvertent rupture of the aneurysm during sternotomy. Adequate decompression of the heart allowed the transfer of the peripheral cannulation to a central aorto-bicaval strategy. Resection of RAAs can also be performed without cardiopulmonary bypass, especially if there are no other intra-cardiac shunts, because there is little risk for systemic thromboembolic phenomena, and if the aneurysms are discrete and small [4]. Most cases of RAAs have had primary closure of the defect, except for some cases where a pericardial patch was used for closure. There is room for initial conservative management if the patient is either unstable or too small in size to allow for safe operation, or in small asymptomatic aneurysms [9]. Other treatment options include using low-dose aspirin thromboprophylaxis and radiofrequency catheter ablation for arrhythmias.

Most cases with right atrial aneurysms have been operated on electively except in one case which necessitated emergency exploration due to cardiac tamponade.

## Conclusion

Large RAAs can be safely operated on using cardiopulmonary bypass with initial peripheral cannulation to decompress the heart, resulting in excellent outcomes.

## Data Availability

Data sharing does not apply to this article as no datasets were generated or analyzed during the current study.

## Abbreviations

RAA: Right atrial aneurysm
CT: Computed tomography
ASD: Atrial septal defect
TEE: Transesophageal echocardiography
E/F: Ejection fraction
CBC: Complete blood count
RFTs: Renal function tests
LFTs: Liver function tests
HIV: Human immunodeficiency virus
CPB: Cardiopulmonary bypass
